# Interactions between cigarette smoking and cognitive status on functional connectivity of the cortico‐striatal circuits in individuals without dementia: A resting‐state functional MRI study

**DOI:** 10.1111/cns.13852

**Published:** 2022-05-04

**Authors:** Tiantian Qiu, Fei Xie, Qingze Zeng, Zhujing Shen, Guijin Du, Xiaopei Xu, Chao Wang, Xiaodong Li, Xiao Luo, Kaicheng Li, Peiyu Huang, Tianyi Zhang, Jinling Zhang, Shouping Dai, Minming Zhang, for the Alzheimer's Disease Neuroimaging

**Affiliations:** ^1^ 529858 Department of Radiology Linyi People's Hospital Linyi China; ^2^ 529858 Department of Equipment and Medical Engineering Linyi People's Hospital Linyi China; ^3^ Department of Radiology The Second Affiliated Hospital of Zhejiang University School of Medicine Hangzhou China; ^4^ 414282 Department of Neurology Tongde Hospital of Zhejiang Province Hangzhou China; ^5^ 529858 Cancer Center Linyi People's Hospital Linyi China

**Keywords:** Alzheimer's disease, functional connectivity, magnetic resonance imaging, smoking, striatum

## Abstract

**Aims:**

Cigarette smoking is a modifiable risk factor for Alzheimer's disease (AD), and controlling risk factors may curb the progression of AD. However, the underlying neural mechanisms of the effects of smoking on cognition remain largely unclear. Therefore, we aimed to explore the interaction effects of smoking × cognitive status on cortico‐striatal circuits, which play a crucial role in addiction and cognition, in individuals without dementia.

**Methods:**

We enrolled 304 cognitively normal (CN) non‐smokers, 44 CN smokers, 130 mild cognitive impairment (MCI) non‐smokers, and 33 MCI smokers. The mixed‐effect analysis was performed to explore the interaction effects between smoking and cognitive status (CN vs. MCI) based on functional connectivity (FC) of the striatal subregions (caudate, putamen, and nucleus accumbens [NAc]).

**Results:**

The significant interaction effects of smoking × cognitive status on FC of the striatal subregions were detected in the left inferior parietal lobule (IPL), bilateral cuneus, and bilateral anterior cingulate cortex (ACC). Specifically, increased FC of right caudate to left IPL was found in CN smokers compared with non‐smokers. The MCI smokers showed decreased FC of right caudate to left IPL and of right putamen to bilateral cuneus and increased FC of bilateral NAc to bilateral ACC compared with CN smokers and MCI non‐smokers. Furthermore, a positive correlation between FC of the NAc to ACC with language and memory was detected in MCI smokers.

**Conclusions:**

Cigarette smoking could affect the function of cortico‐striatal circuits in patients with MCI. Our findings suggest that quitting smoking in the prodromal stage of AD may have the potential to prevent disease progression.

## Graphical abstract

Cigarette smoking might affect cognitive function by modulating functional changes of the cortico‐striatal circuits in individuals without dementia. The effects of cigarette smoking exerted on cortico‐striatal circuits connectivity are different in CN smokers and MCI smokers.

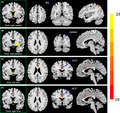

## 1 INTRODUCTION

Alzheimer's disease (AD) is the leading cause of dementia, accounting for about 60%–80% of total dementia cases in the elderly people.^1^, ^2^, ^3^ It has an insidious onset and is clinically characterized by progressive, irreversible cognitive decline. Mild cognitive impairment (MCI) is associated with a high risk of developing a variety of dementias, including AD,^4^ vascular dementia,^5^ and Parkinson's disease dementia.^6^, ^7^ The lack of effective treatment to prevent and reverse the course of AD^8^ highlights the importance of controlling vascular risk factors in the early stage of AD.^3^ Moreover, recent studies have emphasized that vascular dysfunction, such as the white matter hyperintensities^9^ and blood‐brain barrier breakdown,^10^ play a crucial role in AD pathogenesis. Evidence from epidemiological studies and systematic reviews have indicated that cigarette smoking is significantly associated with a higher risk of AD and other forms of dementia, such as vascular cognitive impairment.^11^, ^12^ Therefore, exploring the effects of smoking on brain functional activity in MCI patients is of great significance in guiding early clinical intervention of AD.

Nicotine is the psychoactive component of cigarettes that contributes to the process of smoking addiction. It excites dopaminergic neurons in the mesencephalon by activating the nicotinic acetylcholine receptors(nAChRs) on the dopaminergic somata^13^ to induce the release of dopamine from target regions, particularly the striatum.^14^, ^15^ Dysfunction of the striatal dopamine system, such as decreased dopamine transporter binding^16^ and low dopamine receptors signaling,^17^ has been described in nicotine addiction. To be specific, the striatum is comprised of the dorsal striatum (DS) and ventral striatum (VS). The DS (mainly the caudate and putamen) is involved in motor control and cognitive functions, whereas the VS (mainly the nucleus accumbens [NAc]) is the ventral extension of the DS and plays a central role in reward, the development of addictive behaviors, and habit formation.^18^ Furthermore, the DS and VS showed extensive connections to cortical and limbic regions, such as the prefrontal cortex (PFC), orbital frontal cortex, anterior cingulate cortex (ACC), insula, and hippocampus, which constitute the close cortico‐striatal circuits that contribute to the processes of addiction and cognition.^19^, ^20^ More importantly, recent studies have identified that cortico‐striatal circuits may serve as the potential therapeutic targets for nicotine addiction,^21^, ^22^ which provides insight into the mechanisms of smoking‐induced cognitive alterations in individuals without dementia.

Increasing evidence has suggested that functional connectivity (FC) disruption precedes structural atrophy, reflecting the underlying pathophysiological alterations.^23^, ^24^ Abnormal FC alterations of the cortico‐striatal circuits have been observed in healthy smokers and AD/MCI patients. The FC of the striatum with dorsal ACC in healthy smokers was found to be negatively correlated with the severity of nicotine addiction,^25^ while patients with AD and MCI showed abnormal connectivity between the striatum and cortical regions, including the PFC, medial frontal cortex, and middle temporal cortex.^26^, ^27^, ^28^ Reduced FC of the striatum with precuneus was also found to be correlated with memory decline in patients with AD.^29^ Nevertheless, the exact effects of smoking on FC alterations of cortico‐striatal circuits in MCI patients remains unclear.

This study aimed to explore the interaction effects of smoking × cognitive status based on FC of the striatal subregions. Both DS and VS subregions (caudate, putamen, and NAc) were chosen as the seeds for FC analyses. Based on the role of cortico‐striatal circuits in addiction and cognition, we assume that the functional changes in cortico‐striatal circuits may be associated with the MCI in smoking patients.

## 2 MATERIAL AND METHODS

### 2.1 Study participants

We included 378 cognitively normal (CN) subjects and 182 MCI patients^30^ from the ADNI database (http://adni.loni.usc.edu/). Smoking was defined as the presence of any history of smoking and non‐smoking was defined as participants who reported they never smoked cigarettes during their lifetime (Table S1). After the screening, 49 participants were excluded for excessive head motion (details later). Finally, 304 CN non‐smokers, 44 CN smokers, 130 MCI non‐smokers, and 33 MCI smokers entered subsequent analyses.

### 2.2 Demographics and neuropsychological tests

Demographics including age, sex, education level, and vascular risk factors (hypertension, diabetes mellitus, and hypercholesterolemia) were assessed. Neuropsychological tests involved in multiple cognitive domains, including memory (Wechsler memory scale‐logical memory, WMS‐LM, immediate and delayed recall), attention (Trail‐Making Test, Part A, TMT‐A), execution (Trail‐Making Test, Part B, TMT‐B), and language (Semantic Verbal Fluency, SVF).

### 2.3 Imaging data acquisition

The structural and resting‐state functional MRI (rsfMRI) images of all participants were scanned using a 3.0‐Tesla Philips MRI scanner (Supplementary materials). Details of the ADNI neuroimaging acquisition protocol are publicly available on the Laboratory of Neuroimaging website (http://www.loni.ucla.edu/ADNI).

### 2.4 Preprocessing of rsfMRI and structural MRI data

The rsfMRI data were preprocessed with Data Processing & Analysis for Brain Imaging (DPABI, http://rfmri.org/dpabi), including (a) removal of the first 10 volumes; (b) slice timing; (c) realignment (29 CN non‐smokers, 1 CN smoker, and 19 MCI non‐smokers were excluded due to head motion over 3mm/degree); (d) normalization to the standard EPI template (resampled into 3 × 3 × 3 mm3); (e) smoothing (6 × 6 × 6 mm^3^ full‐width at half maximum Gaussian kernel); (f) linear trends and temporally filter (0.01 Hz < f < 0.08 Hz); (g) regression of nuisance covariates, including Friston 24 motion parameters, global mean signal, white matter (WM) signal, and cerebrospinal fluid (CSF) signal. The mean framewise displacement (FD) of each subject was also calculated.

The T1‐weighted images were preprocessed and analyzed using the Computational Anatomy Toolbox (CAT12, http://dbm.neuro.uni‐jena.de/cat/) and SPM12. The images were bias‐corrected, tissue‐classified (gray matter (GM), WM, and CSF), and registered using linear (12 parameter affine) and non‐linear transformations (warping) within the CAT12 default preprocessing pipeline.

### 2.5 Striatum‐based resting‐state FC analysis

The striatal subregions (caudate, putamen, and NAc) were chosen as the seeds for FC analyses according to Harvard‐Oxford subcortical structural atlas. Dynamic brain connectome (DynamicBC) analysis toolbox (http://restfmri.net/forum/DynamicBC) 31 was used to create individual FC maps by calculating Pearson's correlations between the time course of striatal subregions and whole‐brain areas, which were then transformed into Z maps.

### 2.6 Propensity score matching

Propensity score matching (PSM) implanted in SPSS version 26 was performed to balance the differences in demographic features between non‐smoking and smoking subgroups in CN and MCI. Specifically, a 1: 2 matching was used to pair CN smokers and MCI smokers based on age, sex, and education level. The clinical and striatal FC results were summarized in Tables S2, S3 and Figure S1.

### 2.7 Statistical analysis

The statistical analyses of demographics and neuropsychological data were performed using IBM SPSS 26.0 statistical software. Normal distribution was tested using the Kolmogorov–Smirnov test. One‐way analysis of variance (ANOVA) and Kruskal–Wallis test were used for continuous variables. Pairwise comparisons were Bonferroni‐corrected for four groups. Chi‐square tests were used for categorical variables, including sex and vascular risk factors.

The statistical analyses of FC of the striatal subregions were performed using the DPABI toolbox.^32^ Specifically, we performed a 2 × 2 mixed‐effect analysis to explore the interaction effects of smoking and cognitive status (CN vs. MCI). Age, sex, education level, head motion (FD value), and vascular risk factors were used as covariates. To control the effect of cortical atrophy on the functional analysis, normalized modulated (with the volumetric information encoded) GM maps were used as covariate images. The threshold was set to the voxel level at *p* < 0.005 and the cluster level at *p* < 0.05 after Gaussian random field (GRF) correction. To further understand how smoking and cognitive status interacted on FC of the striatal subregions, we extracted the mean FC values from the interaction regions and further performed *post*‐*hoc* pairwise comparisons (*p* < 0.05, Bonferroni correction). At last, partial correlation analysis was performed to investigate the correlation between the mean FC values of interaction regions and neuropsychological scores with age, sex, and education level as covariates (*p* < 0.05).

## 3 RESULTS

### 3.1 Demographics and clinical characteristics

The demographics and clinical characteristics of the four subgroups were summarized in Table 1. The MCI smokers (76.61±7.54 years) were older (*p* < 0.05) than the CN non‐smokers (72.98±7.25 years). Compared with other subgroups, the CN non‐smokers were predominantly women (*p* < 0.05). There was no significant difference between subgroups in terms of vascular risk factors (*p* > 0.05). The neuropsychological performance of MCI patients (smokers and non‐smokers) was significantly lower than that of CN subjects in memory, attention, execution, and language (*p* < 0.05). There was no significant difference in the neuropsychological performance of smokers (CN and MCI) compared with non‐smokers (*p* > 0.05).

**TABLE 1:** The demographic and clinical characteristics

Variables	CN non‐smokers	CN smokers	MCI non‐smokers	MCI smokers	F/χ^2^	*p*
	(*n* = 304)	(*n* = 44)	(*n* = 130)	(*n* = 33)		
Demographic factors						
Age (years)	72.98 ± 7.25	75.83±7.64	74.33±7.87	76.61±7.54	4.09	0.007^b^
Sex (F:M)	183:121	21:23	56:74	12:21	15.62	0.001
Education (years)	16.88 ± 2.33	16.27±2.61	16.71±2.54	15.79±2.40	2.56	0.054
Vascular risk factors						
Hypertension, n (%)	118 (38.80)	21 (47.70)	59 (45.40)	20 (60.60)	7.04	0.071
Diabetes mellitus, n (%)	7 (2.30)	1 (2.30)	5 (3.80)	0 (0.00)	1.84	0.607
Hypercholesterolemia, n (%)	149 (49.00)	22 (50.00)	73 (56.20)	20 (60.60)	3.01	0.390
Neuropsychological tests						
Memory						
WMS‐LM immediate recall	14.64 ± 3.61	15.23 ± 3.36	10.62 ± 4.30	11.64 ± 4.76	38.55	<0.001^abcd^
WMS‐LM delayed recall	13.53 ± 3.80	14.20 ± 3.62	8.58 ± 4.37	9.61 ± 4.59	54.94	<0.001^abcd^
Attention						
TMT‐A	31.30 ± 9.51	29.84 ± 6.69	36.15 ± 13.05	37.76 ± 12.91	9.96	<0.001^abcd^
Execution						
TMT‐B	74.85 ± 35.90	70.50 ± 27.23	96.23 ± 51.98	110.39 ± 64.58	14.21	<0.001^abcd^
Language						
SVF (animal)	21.68 ± 5.12	21.32 ± 5.87	19.02 ± 5.07	18.67 ± 5.23	10.08	<0.001^ab^
Head Motion (FD value)	0.11 ± 0.07	0.12 ± 0.07	0.11 ± 0.06	0.13 ± 0.07	1.04	0.374

Note

Values are expressed as mean ± standard deviation, number or percentage of participants.

Abbreviations: CN, Cognitively normal; FD, framewise displacement; MCI, Mild Cognitive Impairment; SVF, Semantic Verbal Fluency; TMT, Trail‐Making Test; WMS‐LM, Wechsler memory scale‐logical memory.

^a–d^Post hoc analysis further revealed the source of ANOVA difference (^a^CN non‐smokers vs. MCI non‐smokers; ^b^CN non‐smokers vs. MCI smokers; ^c^CN smokers vs. MCI non‐smokers; ^d^CN smokers vs. MCI smokers) (*p* < 0.05, significant difference between the two groups)

### 3.2 Smoking ×cognitive status interaction on FC of the striatal subregions

**FIGURE 1:**
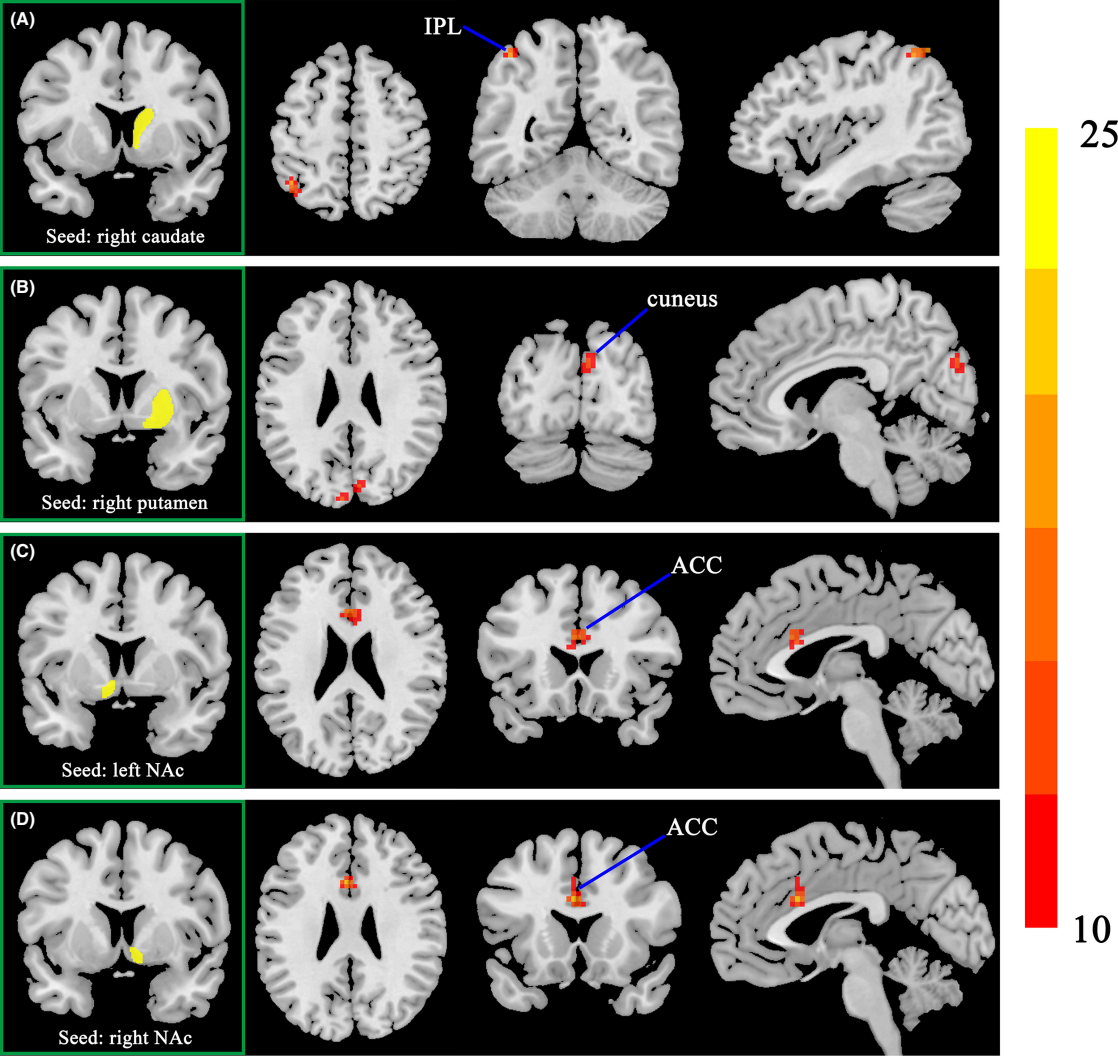
The interaction effects of smoking × cognitive status on FC of the striatal subregions. (A) between right caudate and left IPL; (B) between right putamen and bilateral cuneus; (C) between left NAc and bilateral ACC; and (D) between right NAc and bilateral ACC. IPL, inferior parietal lobule; NAc, nucleus accumbens; ACC, anterior cingulate cortex. The statistical threshold was set at *p* < 0.005 with a cluster‐level of *p* < 0.05 (two‐tailed, GRF corrected)

**FIGURE 2:**
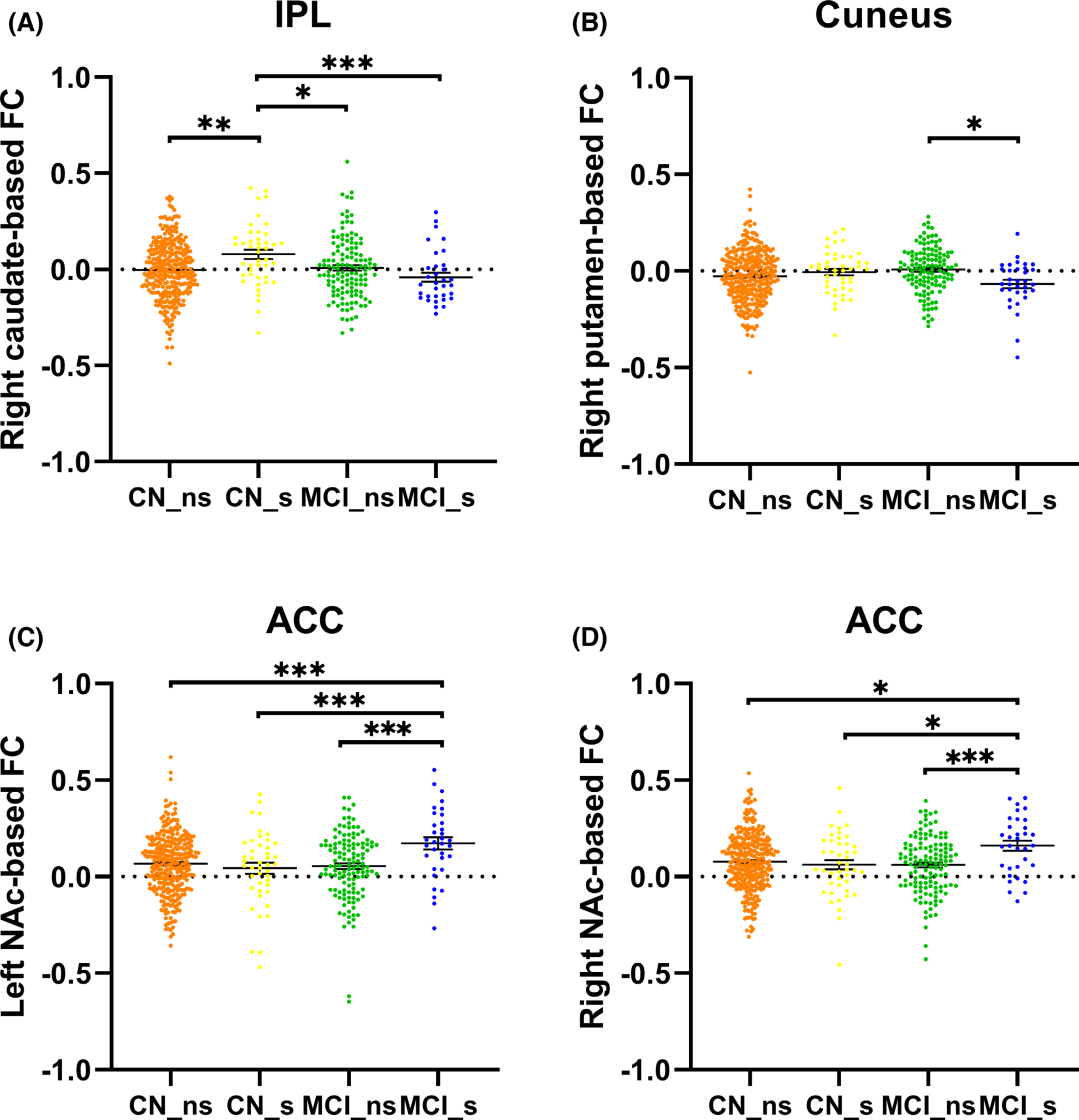
The *post*‐*hoc* analysis of FC values of the interaction regions. The CN smokers showed increased FC of right caudate to left IPL than other subgroups (A). The MCI smokers showed decreased FC of right putamen to cuneus (B) and increased FC of bilateral NAc to ACC (C and D) compared to other subgroups. IPL, inferior parietal lobule; NAc, nucleus accumbens; ACC, anterior cingulate cortex. ^*^ *p* < 0.05, ^**^ *p* < 0.01, ^***^ *p* < 0.005

**FIGURE 3:**
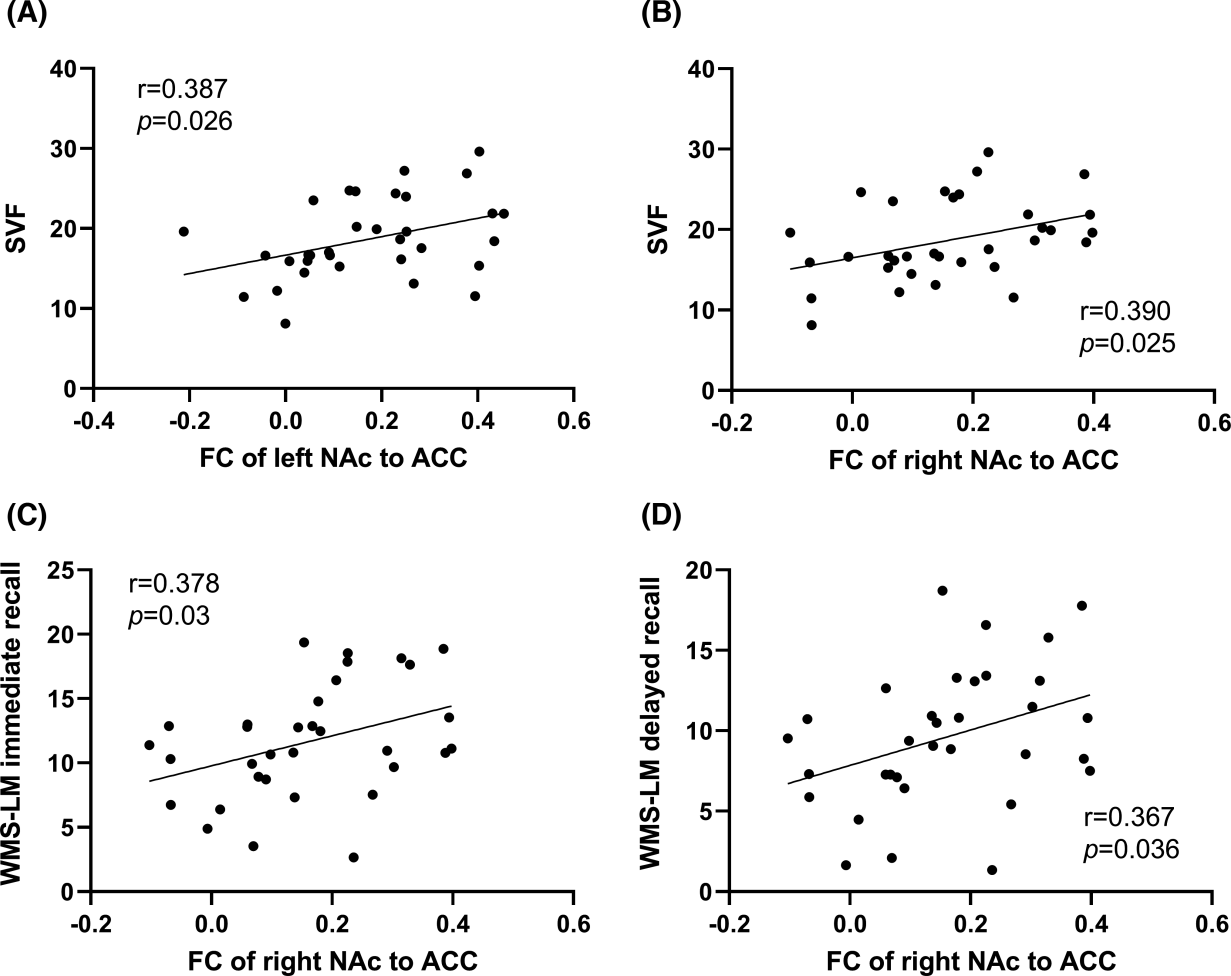
Correlation between FC of the striatal subregions and neuropsychological scores. In MCI smokers, the FC value between left NAc and ACC was positively correlated with language (SVF, r = 0.387, *p* = 0.026); the FC value between right NAc and ACC was positively associated with language (SVF, r = 0.390, *p* = 0.025) and memory (WMS‐LM immediate recall, r = 0.378, *p* = 0.03; delayed recall, r = 0.367, *p* = 0.036). ACC, anterior cingulate cortex; NAc, nucleus accumbens; SVF, Semantic Verbal Fluency; WMS‐LM, Wechsler memory scale‐logical memory

The significant interaction effects of smoking ×cognitive status (CN vs. MCI) on FC of the striatal subregions were detected between: (1) right caudate and left inferior parietal lobule (IPL) (Figure 1A), (2) right putamen and bilateral cuneus (Figure 1B), (3) left NAc and bilateral ACC (Figure 1C), and (4) right NAc and bilateral ACC (Figure 1D). The interaction regions of FC analyses were also summarized in Table 2.

**TABLE 2:** Smoking ×cognitive status interaction on FC of the striatal subregions

Seeds	Interaction effect regions	Peak MNI coordinate			Peak intensity	Cluster voxels
		X	Y	Z		
Right caudate	Left IPL	−42	−54	54	18.5931	21
Right putamen	Bilateral cuneus	6	−81	27	14.368	42
Left NAc	Bilateral ACC	−3	21	24	15.4675	35
Right NAc	Bilateral ACC	−3	18	27	20.4041	28

Note

The statistical threshold was set at *p* < 0.005 with a cluster‐level of *p* < 0.05 (two‐tailed, GRF corrected).

Abbreviations: ACC, anterior cingulate cortex; IPL, inferior parietal lobule; MNI, Montreal Neurological Institute.

Then, *post*‐*hoc* analyses were performed for interaction regions of the four subgroups (Figure 2). Specifically, increased FC of right caudate to left IPL was observed in CN smokers compared with non‐smokers. The MCI smokers showed decreased FC of right caudate to left IPL and of right putamen to bilateral cuneus and increased FC of bilateral NAc to bilateral ACC compared with CN smokers and MCI non‐smokers.

### 3.3 Correlation between FC of the striatal subregions and cognition

We investigated the relationships between FC values of the interaction regions (left IPL, bilateral cuneus, and bilateral ACC) and different cognition domains (Table 3). In MCI smokers, we found that FC value between left NAc and bilateral ACC was positively correlated with language (SVF, r = 0.387, *p* = 0.026), and FC value between right NAc and bilateral ACC was positively associated with language (SVF, r = 0.390, *p* = 0.025) and memory (WMS‐LM immediate recall, r = 0.378, *p* = 0.03; delayed recall, r = 0.367, *p* = 0.036) (Figure 3). After Bonferroni correction (*p* < 0.05/20) for multiple comparisons, there was no significant correlation between FC values of the interaction regions and neuropsychological scores.

**TABLE 3:** Correlations between FC values of interaction regions and neuropsychological scores

	WMS‐LM immediate recall	WMS‐LM delayed recall	TMT‐A	TMT‐B	SVF
Across all groups					
Left IPL	−0.059	−0.025	0.099	0.035	−0.042
Bilateral cuneus	−0.028	−0.019	0.054	0.034	−0.045
Bilateral ACC^a^	−0.024	−0.03	0.098	0.031	0.042
Bilateral ACC^b^	−0.003	−0.007	0.063	0.01	0.003
CN smokers					
Left IPL	0.178	0.115	0.241	0.147	0.096
Bilateral cuneus	−0.229	−0.221	−0.034	0.156	0.14
Bilateral ACC^a^	0.203	0.169	−0.298	−0.177	0.131
Bilateral ACC^b^	0.021	0.062	−0.144	−0.029	−0.129
MCI smokers					
Left IPL	−0.027	0.009	0.078	0.27	−0.096
Bilateral cuneus	0.174	0.167	−0.199	0.11	−0.026
Bilateral ACC^a^	0.145	0.153	0.046	−0.257	0.387*
Bilateral ACC^b^	0.378*	0.367*	0.03	−0.261	0.39*

Note

Data represent correlation coefficients.

Abbreviations: ACC, anterior cingulate cortex; IPL, inferior parietal lobule; SVF, Semantic Verbal Fluency; TMT, Trail‐Making Test; WMS‐LM, Wechsler memory scale‐logical memory.

^a^ Represents FC value between left NAc and bilateral ACC.

^b^ Represents FC value between right NAc and bilateral ACC.

* *p* < 0.05, uncorrected.

## 4 DISCUSSION

In this study, our main goal is to explore the interaction effects of smoking × cognitive status based on FC of the striatal subregions. Our findings showed that regions with interaction effects were primarily located in the IPL, cuneus, and ACC. Specifically, the CN smokers had increased FC between the DS and parietal region compared with non‐smokers, while the MCI smokers showed decreased FC between the DS and parieto‐occipital regions and increased FC between the VS and frontal cortex compared with CN smokers and MCI non‐smokers. Furthermore, FC between the NAc and ACC in MCI smokers was positively correlated with language and memory. This study indicates that cigarette smoking influences the function of cortico‐striatal circuits in patients with MCI.

First, we found that the IPL served as an essential interaction region showing different functional changes in CN smokers and MCI smokers. There is growing evidence that habitual mechanism plays a vital role in addiction.^33^, ^34^ Individuals with higher levels of nicotine addiction have increased engagement of motor preparation circuits, suggesting increased dependence on habitual behavior.^35^ Interestingly, the IPL is the key brain region that controls the conscious motor intention “wanting to move” by specifying a general goal to be reached before movement planning.^36^ Findings from fMRI studies demonstrated smoking cue‐induced activation in the IPL was associated with the severity of nicotine dependence.^37^, ^38^ Thus, our result of higher FC of the caudate to IPL in CN smokers indicates intense motor intentions of smokers on smoking before they smoke.

Besides the function of motor intention, the IPL is also a functional core of the default mode network (DMN) that plays a crucial role in episodic memory retrieval.^39^ Disrupted FC of the IPL/DMN has been widely reported in MCI and AD patients, which is associated with memory decline.^40^, ^41^, ^42^ A progressive decline of structural and functional connections within the DMN has also been observed from CN to MCI and then to AD patients.^43^ To be noted, the accumulation of β‐amyloid (Aβ), which is the pathological hallmark of AD, appears predominantly within the DMN in the early stages.^44^, ^45^ Animal studies also proved that smoking‐related oxidative stress could facilitate Aβ aggregation.^46^ Thus, one possible explanation for lower FC of the caudate to IPL in MCI smokers compared with CN smokers could be that smoking disrupted the cortico‐striatal circuits’ function by exacerbating amyloid pathology. Future longitudinal studies combining amyloid PET and fMRI are needed to verify this speculation. Nevertheless, by combining our findings with previous studies, we assume that the IPL could be a vital interaction region associated with smoking‐related motor intention and cognition decline due to AD.

The interaction effect was also observed between the putamen and cuneus in MCI smokers and non‐smokers. The cuneus is a functional hub of the visual network known to be involved in the integration of visual processing and attention.^47^, ^48^ In a meta‐analysis of fMRI studies, visual areas such as the cuneus, lingual gyrus, and fusiform gyrus showed more reactions to smoking cues than neutral cues, indicating that addicts’ attention was biased toward visual smoking cues through increased activation in the visual cortex.^37^ It has been reported that visual attention deployment has a functional consequence on memory,^49^ and memory decline might stem from the early deteriorations in attention which influences the later memory.^50^ Chronic smoking had a negative influence on cognitive function, including attention and memory,^51^ which are also the common cognitive impairment in the progression of AD.^52^ Meanwhile, evidence from other fMRI studies has consistently elaborated aberrant functional activity in cerebral networks related to the above cognitive domains in MCI and AD patients.^53^, ^54^, ^55^ Taken together, we speculate that smoking affects visual attention through the cortico‐striatal circuits, which further causes memory decline in MCI patients.

In addition, FC between the NAc and ACC was modulated by the smoking ×cognition interactions and correlated with language and memory in MCI smokers. These findings emphasized the role of the fronto‐striatal circuit in information integration between addiction and cognition. The ACC is a part of the brain's limbic system that is involved in various cognitive functions, such as memory retrieval, language preparation, executive control, and visuospatial processing.^56^, ^57^, ^58^ ACC also shows specific interconnections with other PFC and striatal subregions, especially the NAc, constituting the fronto‐striatal reward circuit that plays a key role in nicotine addiction.^59^ Reduced FC of the fronto‐striatal circuit has been observed in cigarette smokers and is negatively correlated with the severity of nicotine dependence.^60^, ^61^ Similarly, in a multicenter large sample study, patients with MCI also showed decreased FC between regions of the fronto‐striatal circuits.^27^ Whereas, we found higher FC between the NAc and ACC in MCI smokers than non‐smokers indicating that smoking might exert some compensatory effects on cognitive impairment, supported by previous work on increased intrinsic brain activity in MCI smokers.^62^ Although smoking is a risk factor for dementia, few studies indicated a protective effect of nicotine on cognition. For example, nicotine can improve working memory, learning, and attention by causing an increased expression or upregulation of α4β2 nAChRs,^63^, ^64^ which are particularly important for modulating cognitive function.^65^ As the smokers in this study include both former and current smokers, the findings might be influenced by the large differences in smoking status. Future longitudinal studies are needed to further expound on the effects of different smoking statuses on cognitive impairment.

Several limitations need to be considered in this study. First, despite the large sample size of the ADNI database, the smoking subgroups are relatively small due to a positive selection from the population in respect to health and lifestyle. Future studies with a larger sample size are needed to verify our work. Second, smoking history in the ADNI database is defined by subjective self‐report from the medical record, including former and current smokers. Most participants lack detailed records, including frequency, number, time of duration, and status (former or current), so further explorations on the effects of different smoking levels or statuses on cognition are necessary. Third, our results of the correlation analysis did not survive after multiple comparison corrections. Still, as an exploratory study, our results could partly reflect the effects of cigarette smoking on cognition in individuals without dementia. Finally, this cross‐sectional study lacks clinical follow‐up to make any possible inference between smoking and AD, longitudinal studies are needed to determine whether the FC alterations of the cortico‐striatal circuits in smokers are related to disease progression.

## 5 CONCLUSION

In this study, we detected the interaction effects of smoking ×cognitive status on FC of the striatal subregions in the IPL, cuneus, and ACC. Our findings indicate that smoking can affect the function of cortico‐striatal circuits, which is associated with cognitive impairment. This study suggests that quitting smoking in the prodromal stage of AD may have the potential to prevent disease progression.

## CONFLICT OF INTEREST

The authors declare that they have no conflict of interest.

## AUTHORS’ CONTRIBUTIONS

Author contributions included conception and study design (TTQ, FX, JLZ, SPD, and MMZ), data collection or acquisition (TTQ, QZZ, ZJS, and GJD), statistical analysis (TTQ), interpretation of results (TTQ, FX, and XPX), drafting the manuscript work or revising it critically for important intellectual content (CW, XDL, XL, KCL, PYH, and TYZ) and approval of final version to be published and agreement to be accountable for the integrity and accuracy of all aspects of the work (All authors).

## ETHICAL APPROVAL

All procedures performed in studies involving human participants were in accordance with the ethical standards of the institutional and/or national research committee and with the 1964 Helsinki declaration and its later amendments or comparable ethical standards.

## CONSENT TO PARTICIPATE

Written informed consent was obtained from all participants and/or authorized representatives and the study partners before any protocol‐specific procedures were carried out in the ADNI study.

## Supporting information

Supplementary Material

## Data Availability

The data used in the preparation of this article were obtained from the Alzheimer's disease Neuroimaging Initiative (ADNI) database: http://adni.loni.usc.edu/.
