# Supplementary material for: Interactions between cigarette smoking and cognitive status on functional connectivity of the cortico‐striatal circuits in individuals without dementia: A resting‐state functional MRI study
Source: CNS Neurosci Ther. 2022 May 4;28(8):1195–204. doi: 10.1111/cns.13852 (PMC9253779; doi:10.1111/cns.13852)
Supplement: Supplementary file 1 — Supplementary Material [file CNS-28-1195-s001.docx]

**Interactions between Cigarette Smoking and Cognitive Status on Functional Connectivity of the** **Cortico-striatal Circuits in Individuals without Dementia:** **A Resting-State Functional MRI Study**

| **Supplementary Table 1A. Smoking information for 44 CN smokers** | | | | | | | | | | | |
| --- | --- | --- | --- | --- | --- | --- | --- | --- | --- | --- | --- |
| Subject | | Description of smoking information | | | | | | |  |  |  |
| 01 | | Smoked 1 pack per day for 15 years | | | | | | |  |  |  |
| 02 | | Smoked 1/2 pack per day for 39 years, and quitted in 1985 | | | | | | |  |  |  |
| 03 | | History of Smoking | | | | | | |  |  |  |
| 04 | | Smoked 1 pack per day for 42 years, and quitted in 1996 | | | | | | |  |  |  |
| 05 | | Smoked 2 packs per day for 4 years, and quitted in 1966 | | | | | | |  |  |  |
| 06 | | Smoked 1/2 pack per month for 20 years, and quitted in 1966 | | | | | | |  |  |  |
| 07 | | Smoked 1 pack or less per day for 18 years, and quitted 1974 | | | | | | |  |  |  |
| 08 | | Smoked for 26 years | | | | | | |  |  |  |
| 09 | | Smoked 1 pack per day for 20 years | | | | | | |  |  |  |
| 10 | | Smoked 1/2 pack per day for 8 years, and quitted in 1974 | | | | | | |  |  |  |
| 11 | | History of Smoking | | | | | | |  |  |  |
| 12 | | History of Smoking | | | | | | |  |  |  |
| 13 | | Pipe and cigarette smoking | | | | | | |  |  |  |
| 14 | | Cigarette smoking | | | | | | |  |  |  |
| 15 | | Pipe and cigar smoking (4-5 pipes/week, 3-4 cigars/week, - both x 20 years) | | | | | | |  |  |  |
| 16 | | Smoked 1 pack per day for 8 years, and quitted in 1961 | | | | | | |  |  |  |
| 17 | | Smoked 1 pack per day for 9 years, and quitted in 1954 | | | | | | |  |  |  |
| 18 | | Smoked 1 pack per day for 4 years, and quitted in 1978 | | | | | | |  |  |  |
| 19 | | Smoked 1.5 packs per day for 20 years, and quitted in 1994 | | | | | | |  |  |  |
| 20 | | Smoked 1 pack per day for 40 years | | | | | | |  |  |  |
| 21 | | Former cigarette smoker | | | | | | |  |  |  |
| 22 | | Former cigarette smoker | | | | | | |  |  |  |
| 23 | | Former cigarette smoker, smoked 1 pack per day for 9 years | | | | | | |  |  |  |
| 24 | | Smoked 1 pack per day for 5 years, and quitted in 1970 | | | | | | |  |  |  |
| 25 | | Smoked for 3 years | | | | | | |  |  |  |
| 26 | | Smoked 1/2 pack per day for 18 years | | | | | | |  |  |  |
| 27 | | History of Smoking | | | | | | |  |  |  |
| 28 | | Former cigarette smoker, smoked 1 pack per day for 20 years | | | | | | |  |  |  |
| 29 | | Smoked 10 cigarettes per day for 8 years, and quitted in 1992 | | | | | | |  |  |  |
| 30 | | Smoked 1 pack per day for 12 years, and quitted in 1972 | | | | | | |  |  |  |
| 31 | | History of Smoking | | | | | | |  |  |  |
| 32 | | History of Smoking | | | | | | |  |  |  |
| 33 | | History of Smoking | | | | | | |  |  |  |
| 34 | | History of Smoking | | | | | | |  |  |  |
| 35 | | History of Smoking | | | | | | |  |  |  |
| 36 | | Smoked cigarettes since his 20's | | | | | | |  |  |  |
| 37 | | History of Smoking | | | | | | |  |  |  |
| 38 | | History of Smoking | | | | | | |  |  |  |
| 39 | | Smoked 5 cigarettes per week | | | | | | |  |  |  |
| 40 | | Smoked 1/2 pack per day | | | | | | |  |  |  |
| 41 | | Cigarette smoking | | | | | | |  |  |  |
| 42 | | Former cigarette smoker | | | | | | |  |  |  |
| 43 | | Smoked 1-2 cigarettes per day | | | | | | |  |  |  |
| 44 | | Former cigarette smoker | | | | | | |  |  |  |
| **Supplementary Table 1B. Smoking information for 33 MCI smokers** | | | | | | | | | |  |  |
| Subject | Description of smoking information | | | | | | | | |  |  |
| 01 | History of Smoking | | | | | | | | |  |  |
| 02 | Smoked for 13 years | | | | | | | | |  |  |
| 03 | Smoked occasional - not even 1/2 pack per day for 2 years, and quitted in 1944 | | | | | | | | |  |  |
| 04 | Smoked 2 packs per day for 24 years, and quitted in 2015 | | | | | | | | |  |  |
| 05 | Smoked for 3 years from 1958 to 1960 | | | | | | | | |  |  |
| 06 | Smoked 1/2 pack per day for 3 years, and quitted in 1972 | | | | | | | | |  |  |
| 07 | History of Smoking | | | | | | | | |  |  |
| 08 | Smoked 4 pipes per day | | | | | | | | |  |  |
| 09 | Smoked 1/2 pack per day for 20 years, and quitted in 1986 | | | | | | | | |  |  |
| 10 | Former cigarette smoker | | | | | | | | |  |  |
| 11 | Cigarette and cigar smoking | | | | | | | | |  |  |
| 12 | Smoked 1/10 pack per day for 11 years | | | | | | | | |  |  |
| 13 | Smoked 3 packs per day for 10 years, and quitted in 1964 | | | | | | | | |  |  |
| 14 | Former cigarette smoker | | | | | | | | |  |  |
| 15 | Smoked 1/2 pack per day for 22 years, and quitted in 1986 | | | | | | | | |  |  |
| 16 | Former cigarette smoker, smoked 1 pack per day for 5 years | | | | | | | | |  |  |
| 17 | Smoked about 2 packs per day in past | | | | | | | | |  |  |
| 18 | Cigarette smoking | | | | | | | | |  |  |
| 19 | Former cigarette smoker, smoked 1 cigarette every other week for about 2 years | | | | | | | | |  |  |
| 20 | Smoked 1 pack per day | | | | | | | | |  |  |
| 21 | Smoked 1 cigar per day | | | | | | | | |  |  |
| 22 | Cigarette smoking | | | | | | | | |  |  |
| 23 | History of Smoking | | | | | | | | |  |  |
| 24 | Smoked 1 packs per day for 40 years | | | | | | | | |  |  |
| 25 | Cigarette smoking | | | | | | | | |  |  |
| 26 | Smoked 2 packs per day for 23 years | | | | | | | | |  |  |
| 27 | Smoked 1.5 packs per day | | | | | | | | |  |  |
| 28 | Smoked pipe | | | | | | | | |  |  |
| 29 | Former cigarette smoker | | | | | | | | |  |  |
| 30 | Cigarette smoking | | | | | | | | |  |  |
| 31 | Cigarette smoking | | | | | | | | |  |  |
| 32 | Cigarette smoking | | | | | | | | |  |  |
| 33 | Cigarette smoking | | | | | | | | |  |  |
| **Supplementary Table 2. The demographic and clinical characteristics.** | | | | | | | | | | | |
| Variables | | | | CN  non-smokers | CN  smokers | MCI  non-smokers | MCI  smokers | F/χ^2^ | *p* | | |
|  |  |  |  | (n=86) | (n=44) | (n=62) | (n=32) |  |  |  |  |
| **Demographic factors** | | | |  |  |  |  |  |  | | |
| Age(years) | | | | 75.14±7.85 | 75.83±7.64 | 76.19±6.83 | 76.19±7.27 | 0.30 | 0.824 | | |
| Sex(F:M) | | | | 40:46 | 21:23 | 22:40 | 12:20 | 2.64 | 0.451 | | |
| Education(years) | | | | 16.16±2.34 | 16.27±2.61 | 16.13±2.47 | 16.03±1.98 | 0.07 | 0.977 | | |
| **Vascular risk factors** | | | |  |  |  |  |  |  | | |
| Hypertension, n(%) | | | | 42(48.8) | 21(47.7) | 29(46.8) | 19(59.4) | 1.50 | 0.682 | | |
| Diabetes mellitus, n(%) | | | | 4(4.7) | 1(2.3) | 3(4.8) | 0(0.0) | 1.98 | 0.576 | | |
| Hypercholesterolemia, n(%) | | | | 49(57.0) | 22(50.00) | 30(48.4) | 19(59.4) | 1.73 | 0.630 | | |
| **Neuropsychological tests** | | | |  |  |  |  |  |  | | |
| Memory | | | |  |  |  |  |  |  | | |
| WMS-LM immediate recall | | | | 14.21±3.94 | 15.23±3.36 | 10.47±3.52 | 11.66±4.83 | 17.85 | *<*0.001^abcd^ | | |
| WMS-LM delayed recall | | | | 12.87±4.16 | 14.20±3.62 | 8.24±3.97 | 9.69±4.64 | 24.92 | *<*0.001^abcd^ | | |
| Attention | | | |  |  |  |  |  |  | | |
| TMT-A | | | | 32.15±9.92 | 29.84±6.69 | 35.02±13.19 | 37.84±13.11 | 4.14 | 0.007^d^ | | |
| Execution | | | |  |  |  |  |  |  | | |
| TMT-B | | | | 83.23±41.46 | 70.50±27.23 | 99.56±58.41 | 104.47±55.76 | 4.92 | 0.002^cd^ | | |
| Language | | | |  |  |  |  |  |  | | |
| SVF(animal) | | | | 20.77±5.01 | 21.32±5.87 | 19.02±4.84 | 18.56±5.28 | 3.13 | 0.027^abcd^ | | |
| Head Motion(FD value) | | | | 0.11±0.07 | 0.12±0.07 | 0.11±0.06 | 0.13±0.07 | 0.91 | 0.435 | | |
| Values are expressed as mean ± standard deviation, number of participants. | | | | | | | | | | | |
| Abbreviation: CN, Cognitively normal; MCI, Mild Cognitive Impairment; WMS-LM, Wechsler memory scale-logical memory; TMT, Trail-Making Test; SVF, Semantic Verbal Fluency; FD, framewise displacement. | | | | | | | | | | | |
| ^a–d^Post hoc analysis further revealed the source of ANOVA difference (^a^ CN non-smokers vs. MCI non-smokers; ^b^ CN non-smokers vs. MCI smokers; ^c^ CN smokers vs. MCI non-smokers; ^d^ CN smokers vs. MCI smokers) (*p*＜0.05, significant difference between the two groups) | | | | | | | | | | | |

| **Supplementary Table 3. Smoking × cognitive status interaction on FC of the striatal subregions.** | | | | | | |
| --- | --- | --- | --- | --- | --- | --- |
| Seeds | Interaction effect regions | Peak MNI coordinate | | | Peak intensity | Cluster voxels |
|  |  | X Y Z | | |  |  |
| Right caudate | Left IPL | -39 | -63 | 57 | 18.5048 | 21 |
| Right putamen | Bilateral cuneus | 6 | -81 | 27 | 15.9433 | 54 |
| Left NAc | Bilateral ACC | -3 | 21 | 24 | 15.6185 | 40 |
| Right NAc | Bilateral ACC | -3 | 18 | 27 | 22.7503 | 49 |
| The statistical threshold was set at *p* < 0.005 with a cluster-level of *p* < 0.05 (two-tailed, GRF corrected). IPL, inferior parietal lobule; ACC, anterior cingulate cortex. | | | | | | |


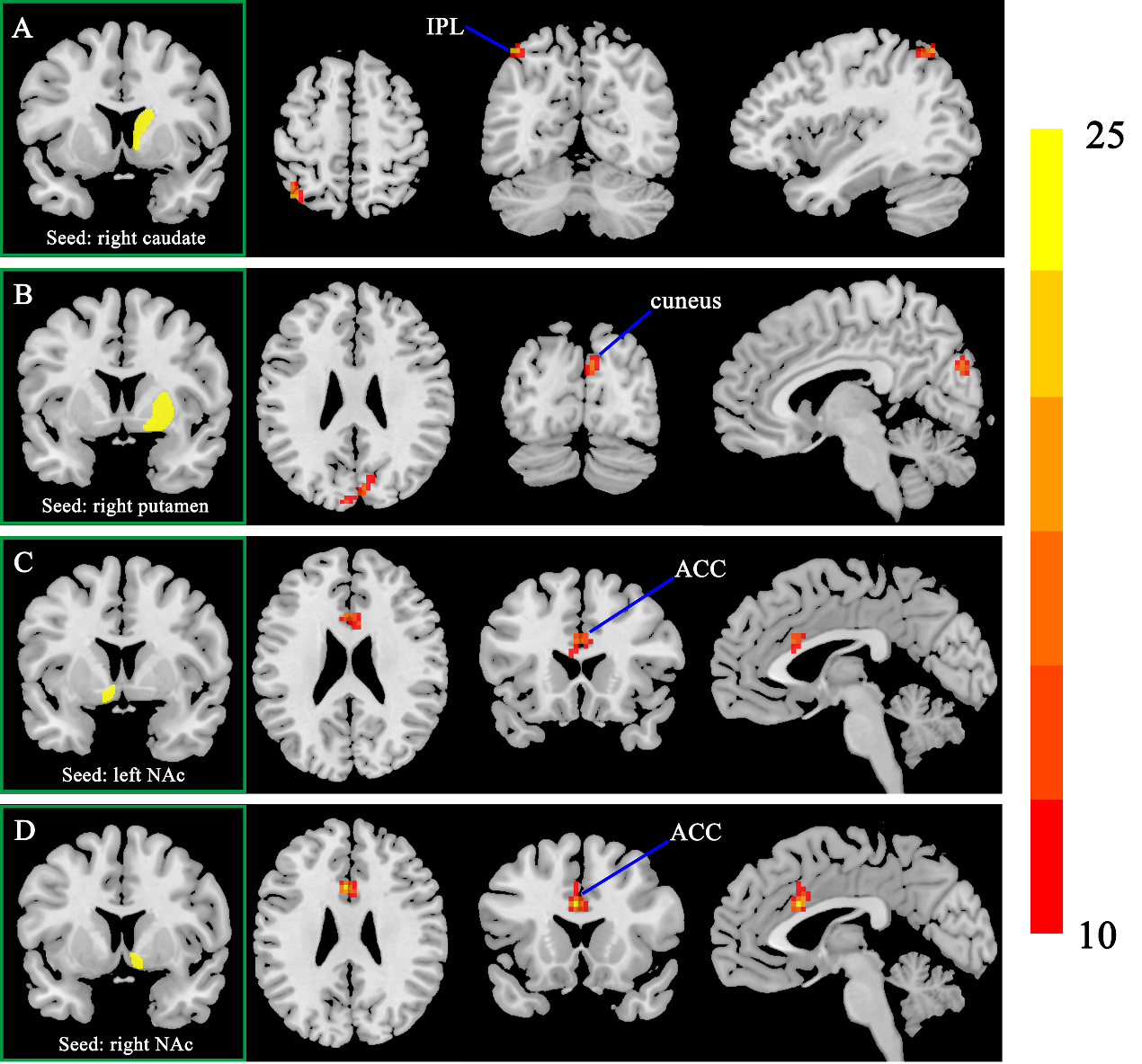


**Supplementary Fig. 1.** The interaction effects of smoking × cognitive status on FC of the striatal subregions after PSM. (A) between right caudate and left IPL; (B) between right putamen and bilateral cuneus; (C) between left NAc and bilateral ACC; and (D) between right NAc and bilateral ACC. IPL, inferior parietal lobule; NAc, nucleus accumbens; ACC, anterior cingulate cortex. The statistical threshold was set at *p* < 0.005 with a cluster-level of *p* < 0.05 (two-tailed, GRF corrected).

**Details of the neuroimaging acquisition protocol:**

The structural MRI images were acquired using a 3D MPRAGE T1-weighted sequence with the following parameters: repetition time (TR) = 2300 ms; echo time (TE) = 2.98 ms; inversion time (TI) = 900 ms; 170 sagittal slices; within plane FOV = 256×240 mm^2^; voxel size = 1×1×1 mm^3^; flip angle = 9^◦^; bandwidth = 240 Hz/pix. The resting-state functional MRI (rsfMRI) images were obtained using an echo-planar imaging sequence with the following parameters: 197 time points; TR = 3000 ms; TE = 30 ms; slice thickness = 3.39 mm; spatial resolution = 3.39×3.39×3.39 mm^3^; flip angle = 90^◦^; and matrix = 64×64.
